# Supplementary material for: Correlation between Phenotype and Genotype in CTNNB1 Syndrome: A Systematic Review of the Literature
Source: Int J Mol Sci. 2022 Oct 19;23(20):12564. doi: 10.3390/ijms232012564 (PMC9604177; doi:10.3390/ijms232012564)
Supplement: Supplementary file 1 [file ijms-23-12564-s001.zip › ijms-1954579-supplementary.pdf]

Table S1. *Spectrum of 84 published CTNNB1 cases*

#	Genetic mutation				Gender	Age (yrs)	Birth measurments			Facial dysmor phism	Eye conditions	Micro-enceph aly	Axial hypotonia/ spasticity	Achieving millestones				Additional clinical features	Source (quality score)
	Exon	Variant	Amino acid change	Mutation type			Lenght (cm)	Weight (g)	OFC (cm)					Sitting (mo)	Crawling (mo)	Walking independetly	Speaking		
1	E3	c.99_100delTG	p.Gly34Asnfs*15	Frameshift	Male	5,5	49	3160	NA	+	Strabismus	+	+/+	14 ; still head-leg	25	6 yrs (cannot stand alone)	Short sentences	Concentration is limited; sensitive to noises, friendly	Kuechler et al. 2015 [2]
2	E3	c.160G>T	p.Glu54*	Nonsense	NA	NA	NA	NA	NA	NA	Strabismus	NA	+/+	NA	NA	Walking with ASO	NA	Dystonia; middle ear effusions	Jin et al. 2020 [5]
3	E3	c.214_215del	p.Gln72fs	Frameshift	Male	5	NA	NA	NA	NA	Normal	+	+/+	NA	NA	NA	Absent speech	GDD, asthma, spastic diplegia	Rossetti et al 2021 [22]
4	E3	c.232C>T	p.Gln78*	Nonsense	Male	11	53	2900	NA	+	Strabismus, hyperopia, astigmatism	-	+/+	NA	Didnt crawl	3 yrs; at 11 yrs coordination problem	Unclear speech; at 11 yrs regression	aggression, anxiety, friendly, sleep problems, stereotypic movements	Winczewska-Wiktor et al. 2016 [23]
5	Intron 3	c.242-1G>C	Splice mutation	Splice	Female	11	NA	NA	NA	NA	Divergent strabismus	-	+/+	NA	NA	NA	NA	NA	Ho et al. 2021 [24]
6	E4	c.274C>T	p.Gln92*	Nonsense	NA	NA	NA	NA	NA	NA	NA	NA	NA	NA	NA	NA	NA	Abnormality of the nervous system	Retterer et al. 2016 [25]
7	E4	c.283C>T	p.Arg95*	Nonsense	Female	4	51	3300	NA	+	Normal	+	+/+	NA	12	4 yrs	Speech apraxia, ~50 words	Aggression when younger, now has stopped	Kuechler et al. 2015 [2]
8	E4	c.283C>T	p.Arg95*	Nonsense	NA	NA	NA	NA	NA	NA	NA	NA	NA	NA	NA	NA	NA	NA	Posey et al. 2017 [26]
9	E4	c. 367 C>T	p.Phe99PhefsTer5	Frameshift	NA	NA	NA	NA	NA	NA	NA	+	+/+	NA	NA	NA	Non-verbal	Bilateral frontal pachygyria; spastic diplegia	Jin et al. 2020 [5]
10	E4	c.400C>T	p.Gln123*	Nonsense	Male	12	NA	NA	NA	NA	Normal	-	+/+	NA	NA	NA	NA	osteogenesis imperfecta, dystonia, blue sclera	Ho et al. 2021 [24]
11	E4	c.423_424insG	p.Tyr142Valfs*4	Frameshift	Female	5,6	51	3450	33	+	Strabismus	+	+/-	NA	24	not yet	Severe, few words (30), sign language	repetitive movements, poor sleeping in infancy	Kuechler et al. 2015 [2]
12	E4	c.493C>T	p.Gln165*	Nonsense	Male	7	NA	NA	NA	NA	Left eye caract, hypermetropia, astigmatism	+	+/+	NA	NA	NA	NA	Dystonia	Ho et al. 2021 [24]
13	E5	c.521; T>C	p.M174T	Missense	Female	34	NA	NA	NA	NA	NA	NA	NA	NA	NA	NA	NA	Severe simple schizophrenia	Levchenko et al. 2015 [27]
14	E5	c.705dupA	p.Gly236Argfs*35	Frameshift	Female	14	NA	2510	32	+	Strabismus	+	+/-	12	NA	4.5 yrs	Babbling at 3yrs, 14 yrs speaking simple sentences, read simple words	Autism	Tucci et al. 2014 [28]
15	Intron 5	c.734+1G>T	Splice mutation	Splice	Female	32	50	3200	33,5	+	Strabismus	+	+/+	2-5 years (40mo)	NA	No	Absent speech	Autism, aggression, ritualistic behaviours, involuntary gestures	Verhoeven et al. 2020 [1]
16	Intron 5	c.734+1G>A	Splice mutation	Splice	Female	49	NA	NA	NA	+	FEVR	NA	+/+	NA	NA	NA	Absent speech	Involuntary rotation of head	Wang et al. 2019 [29]

	Genetic mutation				Gender	Age (yrs	Birth measurments			Facial dysmor phism	Eye conditions	Micro-enceph aly	Axial hypotonia/ spasticity	Achieving millestones				Additional clinical features	Source (data quality assessment)
	Exon	Variant	Amino acid change	Mutation type			Lenght (cm)	Weight (g)	OFC (cm)					Sitting (mo)	Crawli ng (mo)	Walking independetly (yrs)	Speaking		
**17**	Intron 5	c.734+1G>A	Splice mutation	Splicef	Female	27 years	NA	NA	NA	+	FEVR	NA	NA	NA	NA	Walking at 49 yrs (ataxic)	Absent speech	deep tendon hyperreflexia, babinski sign, paroxysmal dystonic HN	Wang et al. 2019 [29]
**18**	E6	c.755delTins AAC	p.Leu252*	Nonsense	Female	15,3	51	2950	33	+	Strabismus, hyperopia	-	+/+	NA	NA	10 yrs	2 words	Dystonia	Kuechler et al. 2015 [2]
**19**	E6	c.776_777de lTC	p.Leu259Pr ofs*11	Frameshift	Male	7	NA	Poor (NA)	NA	NA	mild esotropia, mild hyperopia and astigmatism, strabismus	+	NA/+	NA	NA	NA	NA	Aggresive behaviour, GDD, restless leg, bradykinesia, toe walking, spastic diplegia	Rossetti et al 2021 [22]
**20**	E6	c.796C>T	p.Gln266*	Nonsense	NA	NA	NA	NA	NA	NA	NA	NA	NA	NA	NA	NA	NA	NA	Retterer et al. 2016 [25]
**21**	E6	c.799_809de lGAAGGA GCTAAins GAA	p.Gly268Tr pfsTer5	Frameshift	Female	7	NA	3100	NA	+	NA	+	+/+	18	NA	3 (broad based gait)	No speech	Intermittent loss of skills	Kharbanda et al. 2017 [30]
**22**	E6	c.835delC	p.Leu279Cy sfs*26	Frameshift	NA	NA	NA	NA	NA	NA	NA	NA	NA	NA	NA	NA	NA	NA	Grozeva et al. 2015 [31]
**23**	E7	c.925C>T	p.Gln309*	Nonsense	Male	4,5	NA	NA	NA	+	Hyperopia	+	+/+	18	NA	walking at 4.6 (short distances)	Started speaking at 4 years, but articulation was poor and it was hard to understand	Happy, Feeding difficulties (chewing, swallowing)	Ligt et al. 2012 [4], Tucci et al. 2014 [28]
**24**	E7	c.998dupA	p.Tyr333*	Nonsense	NA	NA	NA	NA	NA	NA	NA	NA	NA	NA	NA	NA	NA	NA	Prasad et al. 2016 [32]
**25**	E7	c.998dupA	p.Tyr333Ter	Nonsense	Female	9	NA	2980	NA	NA	NA	-	+/+	14	NA	4,2 (still had difficulties)	Frst words age 4; more fluent speech age 6; said to be 3 years behind with verbal skills	Violent outburst, dystonia	Kharbanda et al. 2017 [30]
**26**	E7	c.999C> G	p.Tyr333Ter	Nonsense	Female	27 years	NA	2920	NA	+	NA	-	+/+	30	NA	4,5 (ataxic)	First words at 4.5 years; can speak in sentences but speech very unclear	Aggressive behaviour, Asthma; left clubfoot; increased dermatoglyphic whorls	Kharbanda et al. 2017 [30]
**27**	E7	c.999del	p.Tyr333*	Nonsense	Female	15 mo	NA	NA	NA	NA	NA	NA	+/NA	NA	NA	NA	NA	NA	Sun et al. 2019 [33]
**28**	E7	c.1016_1025 delinsT	p.Thr339_A rg342delinsI	Frameshift	Female	3	NA	NA	NA	NA	TRD, strabismus, hyperopia, astigmatism	+	+/NA	NA	NA	NA	NA	GDD, feeding aversion, and poor weight gain, Type-1 Diabetes	Rossetti et al 2021 [22]
**29**	E7	c.1005delA	p.Lys335fs	Frameshift	Male	8	NA	NA	NA	NA	hyperopia, astigmatism, esotropia of the left eye, strabismus	+	+/NA	NA	NA	NA	Absent speech	Aggressive, murmur, GDD, chronic diarrhea, dysplastic+ bicuspid pulmonary valve	Rossetti et al 2021 [22]

	Genetic mutation				Gender	Age (yrs)	Birth measurments			Facial dysmorphism	Eye conditions	Micro-encephaly	Axial hypotonia/ spasticity	Achieving millestones				Additional clinical features	Source (data quality assessment)
	Exon	Variant	Amino acid change	Mutation type			Lenght (cm)	Weight (g)	OFC (cm)					Sitting (mo)	Crawling (mo)	Walking independetly (yrs)	Speaking		
**30**	E7	c.1038_1044 delGCTATCTinsGCT	p.Val349Ala fsTer9	Frameshift	Female	11	NA	2700	NA	+	Strabismus, hypermetropia	+	+/+	NA	13,5	3,5 (ataxic)	Single words at age 5 years, talks in sentences at age 11 years	Stereotypes, aggressive behaviour, acquired teeth early from 3 months	Kharbanda et al. 2017 [30]
**31**	E7	c.1043_1044 delCT	p.Ser348Cys fsX4	Frameshift	Female	8	NA	NA	NA	NA	NA	+	NA	NA	NA	NA	NA	GDD, dystonia	Cordeiro et al. 2018 [34]
**32**	Intron 7	c.1081+1G>C	IVS6 Intron 7	Splice	Male	3,8	47	2720	>2SD	+	Normal	+	+/+	not yet	not yet	not yet	Unclear speech	dystonia; motor restlessness	Kuechler et al. 2015 [2]
**33**	E8	c.1104delT	p.His369Thr fs*2	Frameshift	NA	4	NA	NA	NA	NA	FEVR	+	NA	NA	NA	NA	NA	Autism	Sun et al. 2019 [33]
**34**	E8	c.1105_1109 del	p.His369As pfs*24	Frameshift	Male	22	NA	NA	NA	NA	Divergent strabismus	-	+/+	NA	NA	NA	NA	Dystonia	Ho et al. 2021 [24]
**35**	E8	c.1127G>A	p.Arg376His	Missense	NA	NA	NA	NA	NA	NA	NA	NA	NA	NA	NA	NA	NA	ASD	Krupp et al., 2017 [44]
**36**	E8	c.1163T>C	p.Leu388Pro	Missense	Female	6,8	48	2500	30	+	NA	+	+/+	13	18	2,5	Moderate, first word at 2 ½, 20 words at 4 years but not intelligible	Delayed bone age; swallowing issues	Kuechler et al. 2015 [2]
**37**	E9	c.1251_1252insACGTG	p.Cys419*	Nonsense	Male	15 mo	50	3060	33	NA	NA	+	+/+	not yet	not yet	not yet	Vocalized only a few syllables	Good eye contact, social smile	Kuechler et al. 2015 [2]
**38**	E9	c.1272_1275 del	p.Ser425Thr fs*11	Frameshift	Female	29	NA	NA	NA	+	NA	+	+/+	24	3 years	8 (due to progressive spasticity now with support)	Started speaking first words between 9-10 years; now able to speak a few words	Aggression, automutilaton	Ligt et al. 2012 [4], Tucci et al. 2014 [28]
**39**	E9	c.1272_1275 del	p.Ser425Thr fs*11	Frameshift	Female	3,25	48	2425	NA	+	Strabismus	-	+/+	NA	NA	not yet	Babbles now (aged 3 3/12 years) constantly and some words are understandable	very happy and friendly, but can show a low frustration tolerance, good eye contact	Kuechler et al. 2015 [2]
**40**	E9	c. 1344_1345 InsertionA	p.Arg449Gl nfsTer24	Frameshift	Male	8	NA	NA	NA	NA	Strabismus	+	NA/+	NA	NA	8	First words at 3 years, at 8 years able to speak short sentences	Spastic displegia; dystonia, hyperflexia, dysphagia, hypospadias,	Jin et al. 2020 [5]
**41**	E9	c.1420C>T	p.Arg474*	Nonsense	Male	2	51.5	3830	NA	+	Strabismus	-	+/+	18	No	2	limited words	Sleeping and feeding problems, good eye contact, social	Kuechler et al. 2015 [2]
**42**	E9	c.1420C>T	p.Arg474*	Nonsense	NA	NA	NA	NA	NA	NA	Eye abnormality	NA	NA	NA	NA	NA	NA	NA	Retterer et al. 2016 [25]
**43**	E9	c.1420C> T	p.Arg474Ter	Nonsense	Female	13	NA	3118	NA	+	Strabismus	+	+/+	13	NA	3,5	First words at 4,5 years	Aggressive, ADHD, mouths objects	Kharbanda et al. 2017 [30]

	Genetic mutation				Gender	Age (yrs)	Birth measurments			Facial dysmorphism	Eye conditions	Micro-encephaly	Axial hypotonia/ spasticity	Achieving millestones				Additional clinical features	Source (data quality assessment)
	Exon	Variant	Amino acid change	Mutation type			Lenght (cm)	Weight (g)	OFC (cm)					Sitting (mo)	Crawling (mo)	Walking independetly (yrs)	Speaking		
**44**	E9	c.1420C>T	p.Arg474*	Nonsense	Female	5.25	49	2410	31	+	Strabismus	+	+/+	18	23	not yet	No words	Stereotypic outbursts, feeding problems	Kuechler et al. 2015 [2]
**45**	E9	c.1434_1435insC	p.Glu479Argfs*18	Frameshift	Male	8	NA	NA	NA	+	FEVR and divergent strabismus	+	-/+	NA	NA	NA	NA	NA	Panagiotou et al. 2017 [35]; Ho et al. 2021 [24]
**46**	E9	c.1512G>A	p.Trp504*	Nonsense	NA	NA	NA	NA	NA	NA	NA	NA	NA	NA	NA	NA	NA	Autism	O'Roak et al. 2017 [44]
**47**	E9	c.1543C>T	p.Arg515*	Nonsense	Female	51	NA	NA	NA	+	Optic atrophy	+	+/+	NA	NA	No	Not able to speak, but uses sign language	Progressive swallowing difficulties	Ligt et al. 2012 [4], Tucci et al. 2014 [28]
**48**	E10	c.1603C> T	p.Arg535Ter	Nonsense	Male	3,25	NA	3050	NA	+	Strabismus	+	+/+	8	NA	Unable to walk	Lots of noises but no words	Dystonia	Kharbanda et al. 2017 [30]
**49**	E10	c.1603C> T	p.Arg535Ter	Nonsense	Female	15 mo	NA	2350	NA	+	FEVR	+	+/-	NA	NA	NA	Can make some sounds	NA	Ke et al. 2020 [39]
**50**	E10	c.1603C> T	p.Arg535Ter	Nonsense	NA	NA	NA	NA	NA	NA	NA	NA	NA	NA	NA	NA	NA	NA	Retterer et al. 2016 [25]
**51**	E10	c.1603C> T	p.Arg535Ter	Nonsense	Male	14	NA	3400	NA	+	NA	-	-/+	15	NA	not walking	Moderate; Single words at 14 years	Aggressive, truncal obesity; absent left testis; brachydactyly; achilles tendon contracture	Kharbanda et al. 2017 [30]
**52**	E10	c.1603C>T	p.Arg535*	Nonsense	Male	Died	NA	NA	NA	NA	NA	NA	NA	NA	NA	NA	NA	Dieat at 4 months; abnormal lung growth, pulmonary hypertension	Karolak et al. 2019 [42]
**53**	E10	c.1603C>T	p.Arg535*	Nonsense	Male	NA	NA	NA	NA	NA	NA	NA	NA	NA	NA	NA	NA	NA	Grozeva et al. 2015 [31]
**54**	E10	c.1603C>T	p.Arg535*	Nonsense	Female	15 mo	NA	2350	NA	NA	FEVR, strabismus	+	+/-	Not yet	Not yet	Not yet	Only sounds at 15 months	NA	Ke et al. 2020 [39]
**55**	E10	c.1612C> T	p.Gln538Ter	Nonsense	Female	4,5	NA	2345	NA	+	Strabismus	+	+/+	23	NA	2,5-3 years	First words at 3.4 years	Autism	Kharbanda et al. 2017 [30]
**56**	E10	c.1652C>T	p.Thr551Met	Missense	NA	NA	NA	NA	NA	NA	NA	NA	NA	NA	NA	NA	NA	Autism	O'Roak et al. 2017 [44], Tucci et al. 2014 [28]
**57**	E10	c.1665dupG	p.Thr556fs	Frameshift	Male	4	NA	NA	NA	NA	Retinopathy and strabismus	+	+/+	NA	NA	NA	NA	GDD, ASD, spastic diplegia, aggressive, premature birth (24mo), choreiform movements and intention tremor	Rossetti et al. 2021 [22]
**58**	E10	c.1672C>T	p.Gln558*	Nonsense	Male	15 mo	NA	3600	NA	NA	FEVR	+	+/-	not yet	not yet	not yet	No language at 15 months	Mild thumb adduction	Li et al. 2017 [7]
**59**	E10	c.1677delA	p.Gln559Hisfs*11	Frameshift	NA	NA	NA	NA	NA	NA	NA	NA	NA	NA	NA	NA	NA	Multiple congenital anomalies	Retterer et al. 2016 [25]

	Genetic mutation				Gender	Age (yrs)	Birth measurments			Facial dysmorphism	Eye conditions	Micro-encephaly	Axial hypotonia/ spasticity	Achieving millestones				Additional clinical features	Source (data quality assessment)
	Exon	Variant	Amino acid change	Mutation type			Lenght (cm)	Weight (g)	OFC (cm)					Sitting (mo)	Crawling (mo)	Walking independetly (yrs)	Speaking		
**60**	Intron 10	c.1683+1G>A	Splice mutation	Splice --> nonsense	Female	2,4	51	3630	34,5	+	Normal	+	+/-	not yet	not yet	not yet	Severe, no words; only syllables	At age 2 months she suffered from skin problems (diaper rash and later eczema), aggressive and emotional problems	Kuechler et al. 2015 [2]
**61**	E11	c.1723G>A	p.Gly575Arg	Missense	Female	11	NA	NA	NA	NA	FEVR, legal blind on the right eye, totla retinal detachment on left eye	+	+/+	NA	NA	NA	NA	Repetative behaviours of hand-wringling and nail-picking	Rossetti et al. 2021 [22]
**62**	E11	c.1738_1742 delinsACA	p.Leu580Thrfs*28	Frameshift	NA	NA	NA	NA	NA	NA	FEVR	NA	NA	NA	NA	NA	NA	NA	Sun et al. 2019 [33]
**63**	E11	c.1759C>T	p.Arg587*	Nonsense	NA	NA	NA	NA	NA	NA	NA	NA	NA	NA	NA	NA	NA	NA	Yoo et al. 2017 [40]
**64**	E11	c.1759C>T	p.Arg587*	Nonsense	Female	4	NA	NA	NA	NA	normal	+	+/+	NA	NA	NA	NA	Bicoronal craniosynostosis	Ho et al. 2021 [24]
**65**	E11	c.1801C> T	p.Gln601Ter	Nonsense	Male	6,2	NA	2098	NA	+	FEVR	+	+/NA	NA	NA	not yet	Says Mom and Dad with understanding, uses some Makaton signs, points to the body parts	Sterepotypic and aggressive behaviour, single supernumerary upper incisor; bilateral orchidopexies	Kharbanda et al. 2017 [30]
**66**	E12	c.1849dup	p.Val617Glyfs*4	Frameshift	Male	13	NA	NA	NA	NA	normal	-	+/+	NA	NA	NA	NA	DMD, dystonia	Ho et al. 2021 [24]
**67**	E12	c.1867C>T	p.Gln623*	Nonsense	NA	4	NA	NA	NA	NA	Retinal detachment	NA	NA	NA	NA	NA	NA	NA	Sun et al. 2019 [33]
**68**	E12	c.1923dupA	p.Glu642Argfs*6	Frameshift	Male	8,5	50	2880	31	+	Strabismus, hyperopia	+	+/+	NA	14	8 years	Severe; few single words, gestures	Good social contact, aggressive behaviour, frequent infections, syringomyelia	Kuechler et al. 2015 [2]
**69**	E12	c.1925_1926 del	p.Glu642Valfs*5	Frameshift	Female	14,2	48	3030	NA	+	Strabismus	-	-/+	not yet	NA	No	Moderate, first words at 6 years; not speak in sentences	Autism, short eye contact, tantrums, generalized pyramidal symptoms	Kuechler et al. 2015 [2]
**70**	E12	c.1943A>G	p.N648S	Missense	NA	NA	NA	NA	NA	NA	NA	NA	NA	NA	NA	NA	NA	Severe paranoid schizophrenia	Levchenko et al. 2015 [27]
**71**	E13	c.1962T>A	p.Tyr654*	Nonsense	Male	2	NA	NA	NA	NA	Intermittent exotropia, mild hyperopia, strabismus	+	+/+	NA	NA	NA	NA	GDD, spastic diplegia	Rossetti et al 2021 [22]
**72**	E13	c.1981C> T	p.Arg661Ter	Nonsense	Female	9,2	NA	2530	NA	+	NA	+	+/-	11	NA	2,5	First words at 3,4 years	Obsessional behaviour; Dyspraxia; slightly delayed bone age;	Kharbanda et al. 2017 [30]

	Genetic mutation				Gender	Age (yrs)	Birth measurments			Facial dysmor phism	Eye conditions	Micro-enceph aly	Axial hypotonia/ spasticity Exon	Achieving millestones				Additional clinical features	Source (data quality assessment)
	Exon	Variant	Amino acid change	Mutation type			Lenght (cm)	Weight (g)	OFC (cm)					Sitting (mo)	Crawli ng (mo)	Walking independetly (yrs)	Speaking		
																		hypermobile joints; glue ear	
**73**	E13	c.2038_2041 dup	p.Ser681*	Nonsense	Female	13,2	NA	2523	NA	+	Strabismus, myopia	-	+/+	12	NA	1,5	Mild, full sentences, but delayed	Social, autism, aggressive behaviour, ADHD, scoliosis and a tethered spinal cord	Kuechler et al. 2015 [2]
**74**	E13	c.2038_2041 dup	p.Ser681*	Nonsense	Female	11	NA	2891	NA	+	Strabismus, myopia	+	+/+	12	17	2	Mild, full sentences, but delayed	Communicative, social, aggression, ADHD	Kuechler et al. 2015 [2]
**75**	E13	c.2038_2041 dup	p.Ser681*	Nonsense	NA	NA	NA	NA	NA	NA	NA	NA	NA	NA	NA	NA	NA	Multiple congenital anomalies	Retterer et al. 2016 [25]
**76**	E13	c.2046_2047 del	p.Phe683Gl nfs*9	Frameshift	Male	8 mo	NA	3146	NA	NA	Myopia	NA	NA	NA	NA	NA	NA	NA	Coussa et al. 2020 [36]
**77**	E14	c.2092_2096 dup	p.Ile700Leuf s*37	Frameshift	Female	7	NA	NA	NA	NA	Bilateral FEVR, right convergent strabismus	+	-/+	NA	NA	NA	NA	NA	Ho et al. 2021 [24]
**78**	E14	c.2092_2096 dup	p.Ile700Leuf s*37	Frameshift	Female	37	NA	NA	NA	NA	Normal	-	-/-	NA	NA	NA	Full sentences	NA	Ho et al. 2021 [24]
**79**	E14	c.2112_2116 dup	p.Pro706Gln fs*31	Frameshift	Male	22 mo	NA	1842	NA	NA	FEVR	NA	NA	NA	NA	NA	NA	ASD	Dixon et al. 2016 [37]
**80**	E14	c.2128C>T	p.Arg710Cy s	Missense	inherite d	NA	NA	NA	NA	normal	FEVR	NA	Normal	Normal	normal	normal	normal	NA	Panagiotou et al. 2017 [35]
**81**	E15	c.2142_2157 dup16	p.His720*	Nonsense	inherite d	NA	NA	NA	NA	normal	FEVR	NA	Normal	Normal	normal	normal	normal	NA	Panagiotou et al. 2017 [35]
**82**	E15	c.2273del	p.His758Le ufs*30	Frameshift	Male	14	NA	NA	NA	NA	NA	-	NA	NA	NA	NA	NA	Autism, severe ID	Thevenon et al. 2016 [38]
**83**	Entire gene	333 kb incl. entire gene & ex. 35-37 of ULK4	Gross del	None	Female	5,2	43,5	2100	30,5	+	Hyperopia	+	+/-	14	NA	4,5 years (ataxic)	At 4.5 years could combine several words, count to 10	Friendly, social, short focus, orofacial dyspraxia; sacral dimple	Dubruc et al. 2014 [41]
**84**	Entire gene	505 kb incl. entire gene	Gross del	None	Male	3	45,5	2107	29,5	+	Esotropia	+	+/-	NA	not yet	not yet	Babbles and say “mama” and “dada” specifically shortly before age 3 years	Happy, good eye contact, dystonic movement	Kuechler et al. 2015 [2]
